# A new tunable bandstop filter square-ring resonator using varactor diodes

**DOI:** 10.1371/journal.pone.0290979

**Published:** 2023-09-01

**Authors:** José Garibaldi Duarte Júnior, Valdemir Praxedes da Silva Neto, Adaildo Gomes d’Assunção

**Affiliations:** Department of Communication Engineering, Federal University of Rio Grande do Norte, Natal, RN, Brazil; Edinburgh Napier University, UNITED KINGDOM

## Abstract

This work presents the development of a bandstop filter with a tunable response. Varactor diodes are used as control elements. Studies and investigations demonstrate the influence of the variable capacitance on the input admittance and on the S-parameters frequency responses of the proposed square-ring resonator geometry. The design of the square-ring resonator is based on mathematical modeling of ideal transmission lines, considering parameters of characteristic admittance and electrical length for odd and even excitation modes. Based on S-parameters in ports, an equivalent circuit model of the resonator geometry is presented. The corresponding results are compared with numerical simulations. Comparative analyses are presented in order to guide the process of optimizing the physical dimensions of the layout. A prototype with dimension 0.0272 $\lambda_{g}^{2}$ was designed, fabricated, and tested. As a measured result, a filter with two rejection bands was obtained, the first at 0.6–1.15 GHz and the second at 1.71–2.28 GHz, with 63.0 and 29.0% tuning range, respectively. In comparison with bandstop filters from the literature, the proposed reconfigurable filter presents a larger tuning range for the first band, sufficient inband rejection levels for several applications, and reduced physical dimensions. The proposed configuration is an attractive reconfigurable filtering device for use in modern communication systems operating below 3.0 GHz.

## 1. Introduction

Over the last decades, new communication technologies have required microwave devices with an increasingly specific response [1]. Filters are elements of the communication chain that act directly in the process of accepting or rejecting bands of the frequency spectrum, thus playing a crucial role [2]. The bandstop filters (BSF) class works incessantly on the front-end of radiofrequency systems in the process of rejecting unwanted signals. The last generations of wireless communication stimulate the development of microwave filters with characteristic requirements of operating frequency, bandwidth, external quality fac-tor, in-band and out-of-band attenuation and other requirements [3].

Recently, several models of filters with wideband and multiband frequency response have been developed [4–7]. It is common to use coupled line topologies [8], multiple transmission poles and zeros [9], stepped-impedance resonators (SIR) [10] and classical geometries (square/split-ring) [11]. In [12] a bandstop filter with high frequency selective response is presented. The model is based on a set of symmetrical coupled lines to obtain a high selectivity in the band below 1.0 GHz, however a prototype with considerable dimensions is obtained. In [13, 14] bandstop filters with wideband response are proposed. Coupled lines are used in [13] to obtain multiple transmission poles to increase the rejection range. In [14] an L-shaped resonator is presented together with its coupled line modeling. A bandstop filter is used to improve the isolation of a MIMO antenna in [15]. Its architecture is based on the classic split-ring resonator geometry.

As a consequence of the advent of modern communication systems, microwave devices with reconfigurable response are gaining more and more attention due, among other reasons, to provide an efficient use of the frequency spectrum [16, 17]. Tunable microwave filters can replace a number of other components through their reconfigurability and adaptability without the need to change the design. According to the literature, frequency tuning can be based on the use of ferroelectric capacitors [18], PIN diodes [19], and more recently varactor diodes [11, 20–23]. A reconfigurable bandstop filter based on the use of varactor diodes has the ability to adjust the stop band, and other related parameters, in a continuous way over a delimited frequency band, unlike models with PIN diodes. and microelectromechanical switches (MEMs) whose response is based on discrete and limited states of operation [2]. On the other hand, it is common to need RF-DC polarization and filtering circuits to integrate the reconfiguration element together with the filter geometry, requiring additional studies and analysis, thus increasing the complexity of the design.

In [20, 22, 24] bandstop filter models with multiple operating modes are proposed. PIN diodes and varactors are used in conjunction with planar lines to achieve response adjustment at frequency in different modes. However, there is a considerable increase in the complexity of the design, construction, and the prototypes have large physical dimensions. The filter models proposed in [21, 23] describe a unit element formed by a planar transmission line coupled to a varactor diode and its respective feed-polarization circuit. Given this, it is possible to optimize the element performance and combine it with other planar filtering structures. The models obtained demonstrate considerable insertion and reflection losses in the transmission and rejection bands, respectively. In [25] the use of a set of PIN diodes is proposed to obtain a wideband response from a bandstop filter. However, the final model has excessive DC polarization inputs, which makes it difficult to operate in various applications. A defected ground-based model of varactor diodes is used to obtain a wideband variable response in [26]. In [27, 28] are proposed multimode bandstop filters based on SAW resonator structures and mechanical switches. In [11] a filter model integrated with the feed line of a planar antenna is presented. This is based on the use of a Split-Ring Resonator (SRR) in the geometry and varactors diode. It is common to use square-ring geometries in bandpass filters [6, 29], resulting in models with large dimensions and fixed frequency response. New uses of this classic geometry together with elements to promote reconfiguration functionalities in the development of other microwave filtering classes are promising.

This work aims to develop a bandstop filter with reconfigurable frequency response based on the use of varactor diodes. As an application example, a prototype with a tunable response below 3.0 GHz is presented to be used in modern communication systems [2]. The proposed Tunable Bandstop Filter (TBSF) model is based on the classical square-ring resonator (SRR) geometry. Studies are presented in order to understand and optimize the filter layout in terms of its input admittance (*Y*_*in*_), Tuning Range (*Rf*) and external quality factor (*Q*_*e*_). Also, the presence of a variable capacitance is considered as an element of reconfiguration of the frequency response throughout the studies. Numerical simulations are obtained with a full-wave software tool, as well as the experimental results of the fabricated prototype. A good agreement is verified between these results, thus validating the proposed design procedure. The article begins with the presentation of the TBSF model, followed by its application to the study of the input admittances. After a detailed analysis and discussion, the final model is proposed. Finally, simulation results obtained with the proposed model are compared with the prototype’s experimental results.

## 2. Tunable bandstop filter design

**Fig 1:**
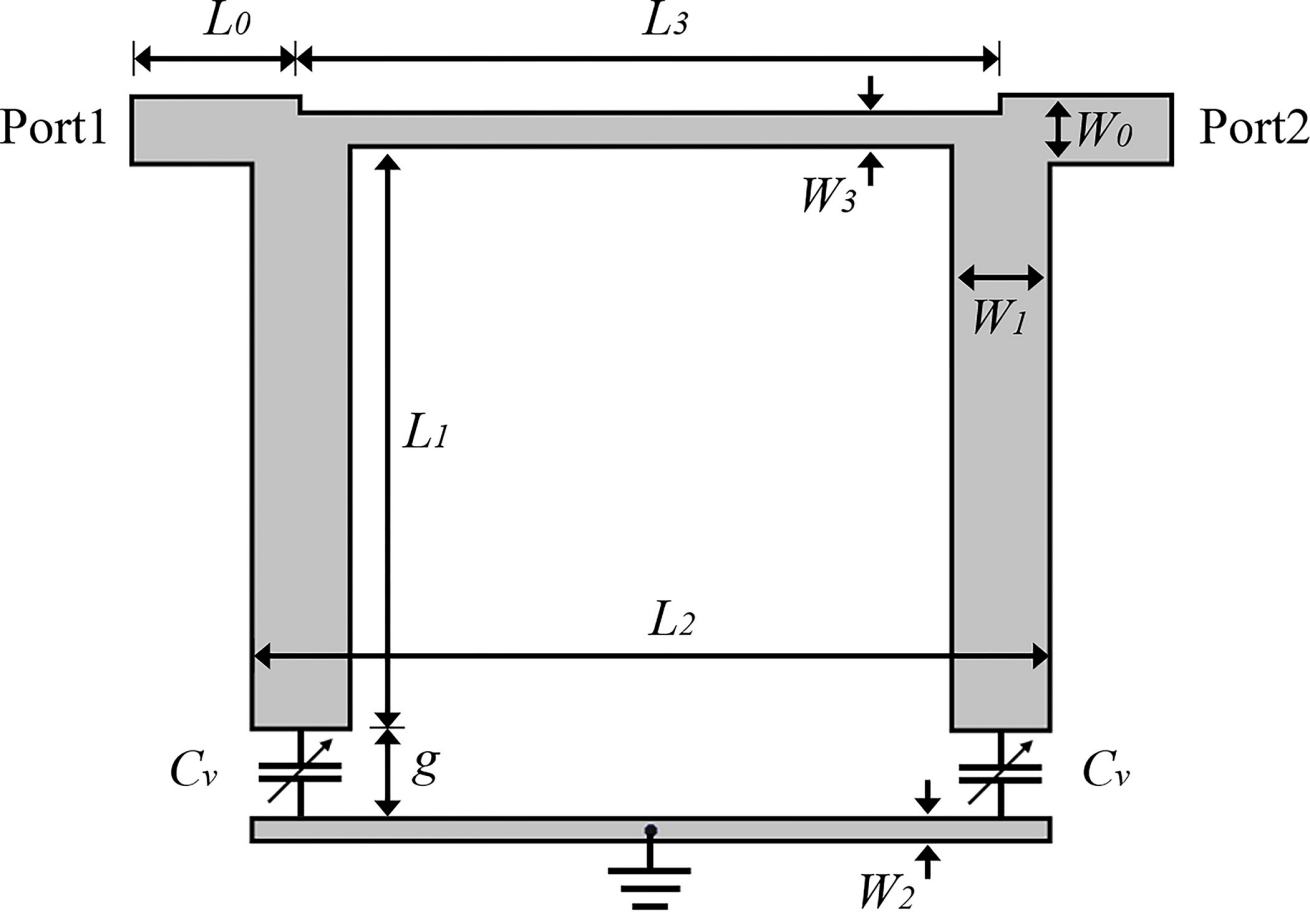
TBSF layout with its physical parameters.

Fig 1 shows the proposed planar tunable bandstop filter (TBSF) layout. Its geometry resembles the classic square-ring resonator structure, also discussed in [6] but with the proposal of a bandpass filter. The dimensions of the edges of the geometry are defined ideally as being of electrical length *θ*_0_ equal to *λ*_0_/4, with *λ*_0_ representing the guided wave-length at the resonance frequency *f*_0_. Along the rectangular geometry, two planar gates designed to present an input impedance of 50.0 Ω at *f*_0_ are inserted for the connection of the input and output of the filter. The insertions of gap g in the geometry, where the varactor diodes symbolized by *C*_*v*_ are inserted, are highlighted, and a short circuit centered on the lower edge of the rectangular geometry connecting the metallization of the geometry in the upper part of the dielectric with the ground plane in the lower region. The planar layout is placed on a RO3006 dielectric substrate with thickness h 1.52 mm, relative permittivity *ε*_*r*_ 6.15 and loss tangent 0.0024. The other dimensions of the geometry are shown in Fig 1. In the next section, a study is presented regarding the modeling of the resonator and its frequency response in terms of its input admittance considering the variable capacitance *C*_*v*_. An analysis in terms of the admittance/impedance parameters of the microstrip lines is developed in order to complement the study of the frequency response of the TBSF layout and to optimize the physical parameters of the geometry.

### 2.1 Study of input admittances

**Fig 2:**
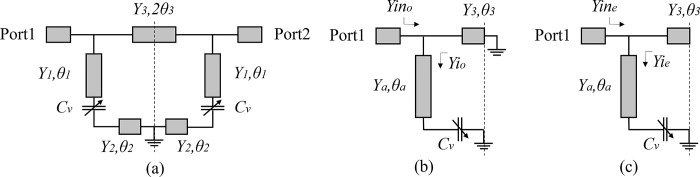
(a) Circuit model of the proposed TBSF layout. (b) Odd-mode circuit. (c) Even-mode circuit.

Fig 2(A) presents the proposed layout model for the TBSF geometry in terms of equivalent circuit represented by the characteristic admittance *Y*_*i*_ of each line portion and its respective electrical length represented by *θ*_*i*_ (i = 1, 2, 3). To analyze the model, the study of input admittances was adopted considering odd and even excitation modes [30]. For this, the equivalent excitation circuits represented in Fig 2(B) and 2(C), odd and even, respectively, are considered. In order to simplify the study, preserving its answer, some modifications are considered in relation to the circuit of Fig 2(A). The variable capacitances symbolized by *C*_*v*_ are replaced by the admittances *Y*_2_. Admittances *Y*_1_ and *Y*_2_ are grouped into a new admittance *Y*_*a*_, where *Y*_*a*_ = *Y*_1_ = *Y*_2_ and *θ*_*a*_ = *θ*_1_+*θ*_2_, as shown in Fig 2(B) and 2(C).

According to [30], the input admittances of a line terminated in short-circuit (s.c.) and in open circuit (o.c.) can be represented as follows, respectively:

$$s.c.\to Y_{in}=\frac{Y_{i}}{j\;\tan\;\theta_{i}}$$ (1)

$$o.c.\to Y_{in}=jY_{i}\;\tan\;\theta_{i}$$ (2)

Applying these conditions to the case of the *Y*_*ino*_ odd-mode input admittance of the circuit in Fig 2(B), we have:

$$Y_{ino}=Y_{io}+\frac{Y_{3}}{j\;\tan\;\theta_{3}}$$ (3)

$$Y_{io}=\frac{jY_{a}\omega C_{v}+jY_{a}^{2}\;\tan\;\theta_{a}}{Y_{a}-\omega C_{v}\;\tan\;\theta_{a}}$$ (4)

$$Y_{ino}=\frac{-Y_{a}(\omega C_{v}\;\tan\;\theta_{3}+A\;\tan\;\theta_{3}-Y_{3})-Y_{3}\omega C_{v}\;\tan\;\theta_{a}}{j(Y_{a}\;\tan\;\theta_{3}-\omega C_{v}C)}$$ (5)

Doing similarly for the circuit in Fig 2(C), which represents the even-mode input admittance, we have:

$$Y_{ine}=Y_{ie}+jY_{3}\;\tan\;\theta_{3}$$ (6)

$$Y_{ie}=Y_{a}\frac{j\omega C_{v}+jY_{a}\;\tan\;\theta_{a}}{Y_{a}+j(j\omega C_{v})\;\tan\;\theta_{a}}$$ (7)

$$Y_{ine}=\frac{jY_{a}(\omega C_{v}+A+B)-jY_{3}\omega C_{v}C}{Y_{a}-\omega C_{v}\;\tan\;\theta_{a}}$$ (8)

where the variables *A*, *B* and *C* are equal to:

$$A=Y_{a}\;\tan\;\theta_{a};B=Y_{3}\;\tan\;\theta_{3};C=\;\tan\;\theta_{a}\tan\theta_{3};$$ (9-11)

It is important to highlight the presence of the variable capacitance *C*_*v*_ in both expressions (5) and (8), characterizing the total dependence of the input admittances in relation to a variable capacitance present in the circuit. The *ω*, which represents the characteristic angular frequency, can be rewritten as 2*πf*_0_. The electrical lengths *θ*_*i*_ can be rewritten as [21]:

$$\theta_{i}=\beta l_{i}=\frac{\sqrt{\varepsilon_{ref}}∙2\pi f_{0}}{c}l_{i}$$ (12)

where *ε*_*ref*_ is the effective dielectric constant, *β* is the propagation constant and *c* is the speed of light in the medium.

**Fig 3:**
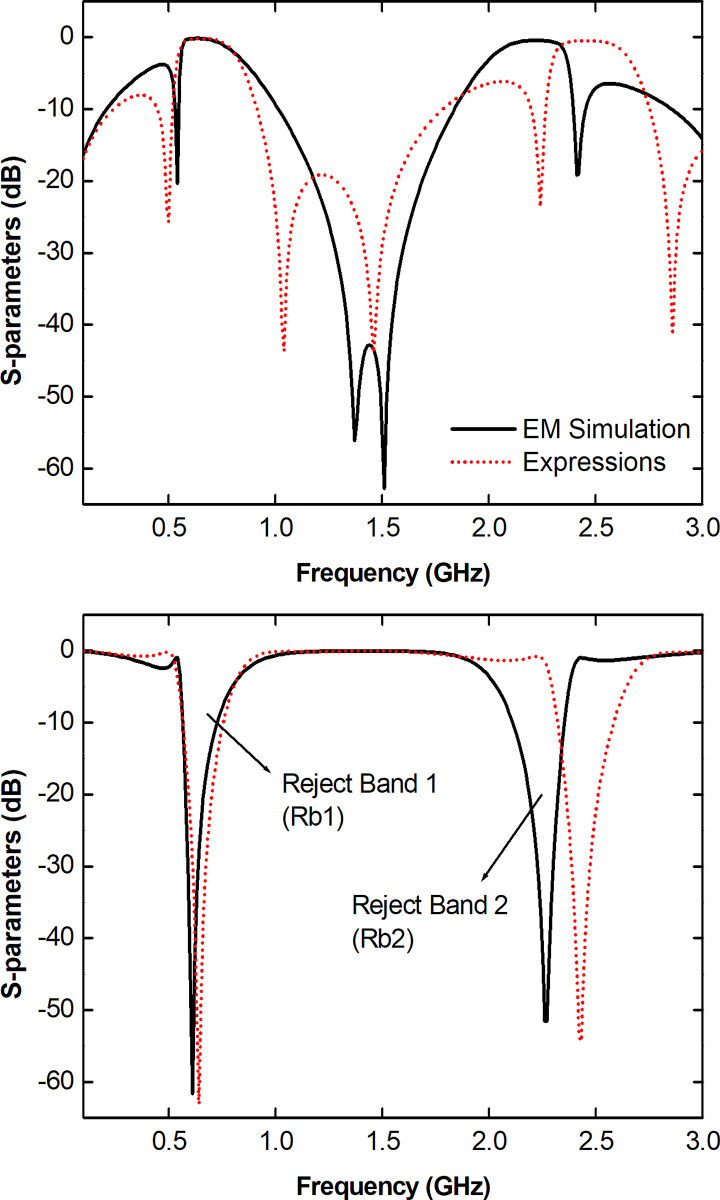
TBSF frequency response. (a) S_11_. (b) S_21_.

Considering the classical theory of transmission lines and their excitation modes [30, 31, 32], and using expressions (5), (8) and (12), it is possible to establish the transformation matrices and obtain the frequency response for the reflection (S_11_) and transmission (S_21_) scattering parameters on the ports of the layout based on the admittances of each mode. Considering a RO3006 dielectric substrate with *ε*_*r*_ = 6.15, tan *δ* = 0.0024, h = 1.52 mm, *Z*_1_ = *Z*_2_ = *Z*_*a*_ = 90.0 Ω (*Y*_*a*_ = 0.0111 S), *Z*_3_ = 50.0 Ω (*Y*_3_ = 0.02 S), *f*_0_ = 0.60 GHz and a *C*_*v*_ capacitance of 4.0 pF, Fig 3 shows the typical frequency responses for the TBSF model obtained via numerical simulation in the ADS circuit analysis software and calculation based on the development of expressions (5) and (8).

Fig 3(A) shows the reflection coefficient on port 1. In Fig 3(B) the transmission coefficient between ports 1 and 2 of the model is observed. It is possible to notice two rejections frequency bands within the analyzed spectrum considering a rejection level higher than -3.0 dB: Rb1 between 0.55 and 0.85 GHz (49.18% of FBW), and Rb2 between 1.98 and 2.38 GHz (17.69% of FBW) for the curves referring to numerical EM simulation. In comparison, the simulated and calculated analytically responses are similar, but with a small frequency shift in Rb2 of 170.0 MHz.

### 2.2 Frequency response analysis

This subsection analyzes the frequency response of the TBSF as a function of the variable capacitance *C*_*v*_ and other physical parameters of the resonator geometry. For this, two key parameters are adopted in the development of tunable filters: Tuning Range (*Rf*) of *f*_0_ given in expression (13) [2,3] and the external quality factor (*Q*_*e*_). *Rf* represents the filter’s operating amplitude as a function of the adjustment factor, in this case, the variable capacitance *C*_*v*_. *f*_0*h*_ and *f*_0*l*_ represent the highest and lowest frequency, respectively, with |S_21_| below -3.0 dB considering the *C*_*v*_ variation. *Rf* allows, among other considerations, to express how much the filter response can vary along the frequency spectrum. The external quality factor *Q*_*e*_ describes the relationship between the resonant frequency f_0_ and the bandwidth (BW) of operation commonly measured in |S_21_| equal to -3.0 dB.


$$Rf=\frac{f_{0h}-f_{0l}}{\frac{\left(f_{0h}+f_{0l}\right)}{2}}$$ (13)

**Fig 4:**
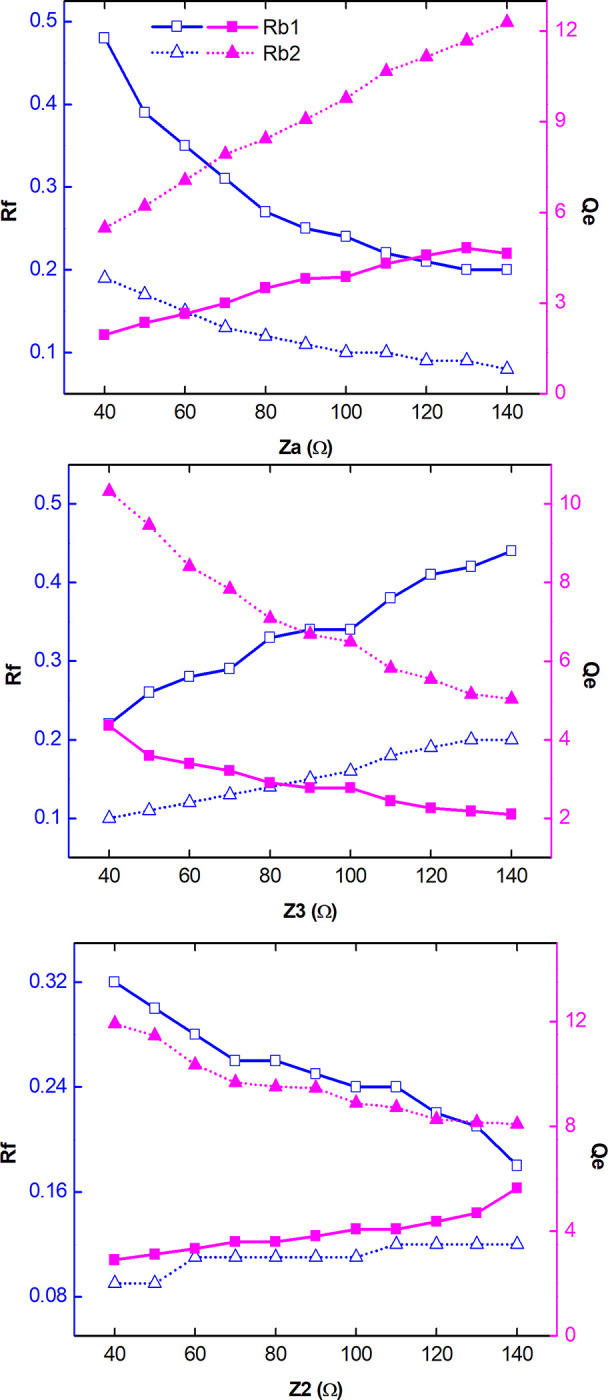
TBSF analysis in function of (a) *Z*_*a*_, (b) *Z*_3_ and (c) *Z*_2_ in Ω.

Tuning Range (*Rf*) and external quality factor (*Q*_*e*_) parameters were investigated for both rejection bands, Rb1 and Rb2. Initially the frequency response of *Rf* and *Q*_*e*_ was analyzed as a function of the impedance *Z*_*a*_ = 1/*Y*_*a*_ and the variation of *C*_*v*_ between 0.72 and 4.15 pF, as shown in Fig 4(A). The other physical parameters are the same ones used in Fig 3. There is a decrease in *Rf* (blue curves) in both rejection bands, being more pronounced for Rb1. There is an increase in *Q*_*e*_ (magenta curves) for the two TBSF rejection bands due to the increase in *Z*_*a*_, thus representing a higher filter selectivity under these conditions. For impedance *Z*_3_ = 1/*Y*_3_, the behavior of *Rf* and *Q*_*e*_ are inverted, as shown in Fig 4(B). The increase in *Z*_3_ provides a greater tuning amplitude for the filter (*Rf*), while causing a decrease in *Q*_*e*_, in both TBSF rejection bands. For *Z*_2_ = 1/*Y*_2_, the increase in impedance causes a decrease in *Rf* and an increase in *Q*_*e*_ for Rb1. As for Rb2, there is a slight increase in *Rf* and a decrease in *Q*_*e*_ levels, as shown in Fig 4(C).

**Fig 5:**
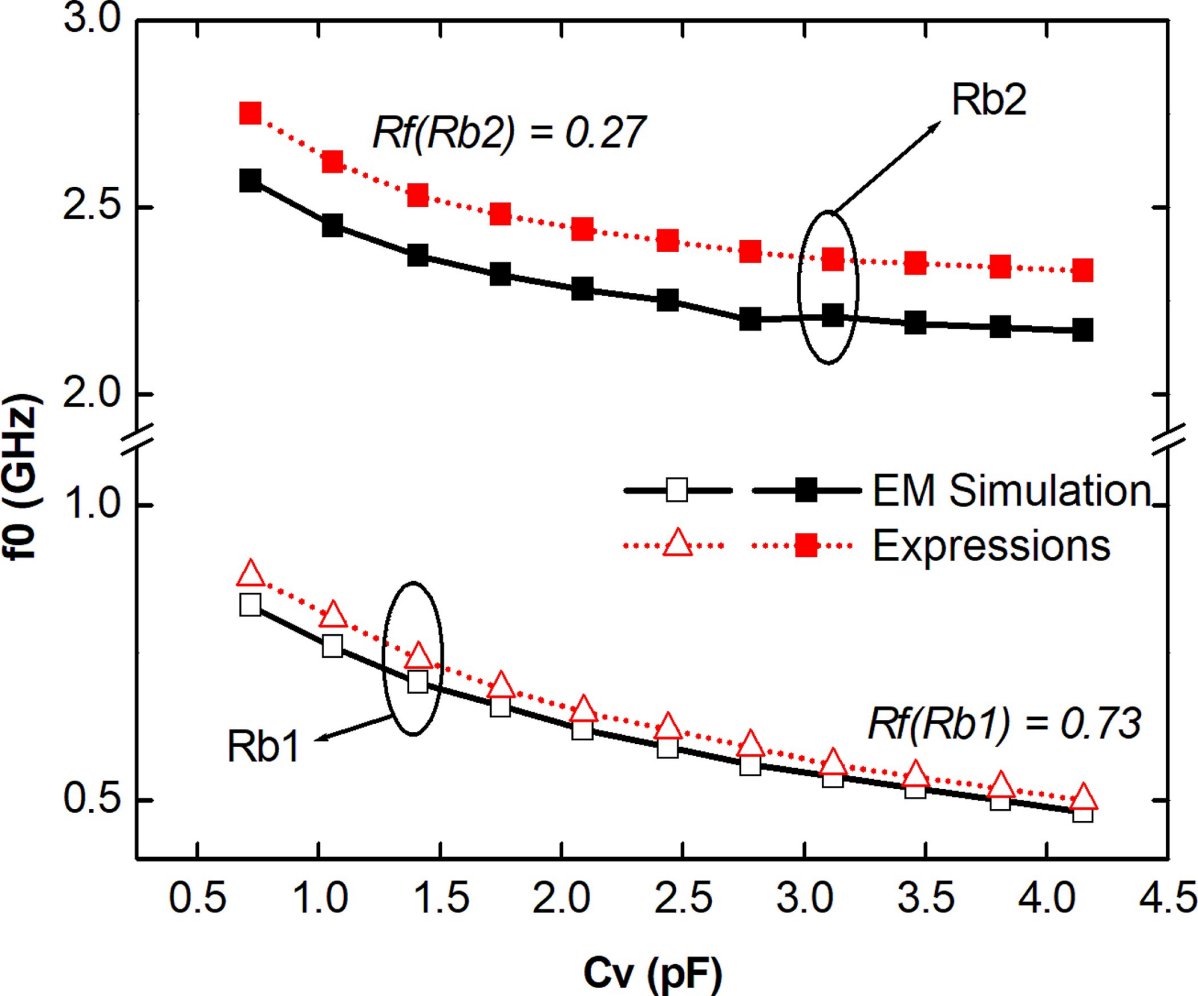
Resonance frequencies f_0_ (GHz) as a function of variable capacitance Cv (pF), for Rb1 and Rb2.

Fig 5 shows the results, obtained by simulation and analytically, of the rejection frequencies f_0_ for both bands (Rb1 and Rb2) as a function of the variable capacitance *C*_*v*_. For both Rb1 and Rb2, a negative displacement of the rejection frequencies is verified due to the increase of the variable capacitance *C*_*v*_. For the first rejection band (Rb1), an offset between 0.48 and 0.83 GHz with |S_21_| below -3.0 dB resulting in a Tunning Range *Rf* of 0.73. For Rb2, an *Rf* of 0.27 is observed, with an offset between 2.17 and 2.57 GHz as a function of the variable capacitance *C*_*v*_. It is then observed for Rb1, an amplitude of 350 MHz and for Rb2, 400 MHz are obtained given the variable tuning of the proposed TBSF model.

**Fig 6:**
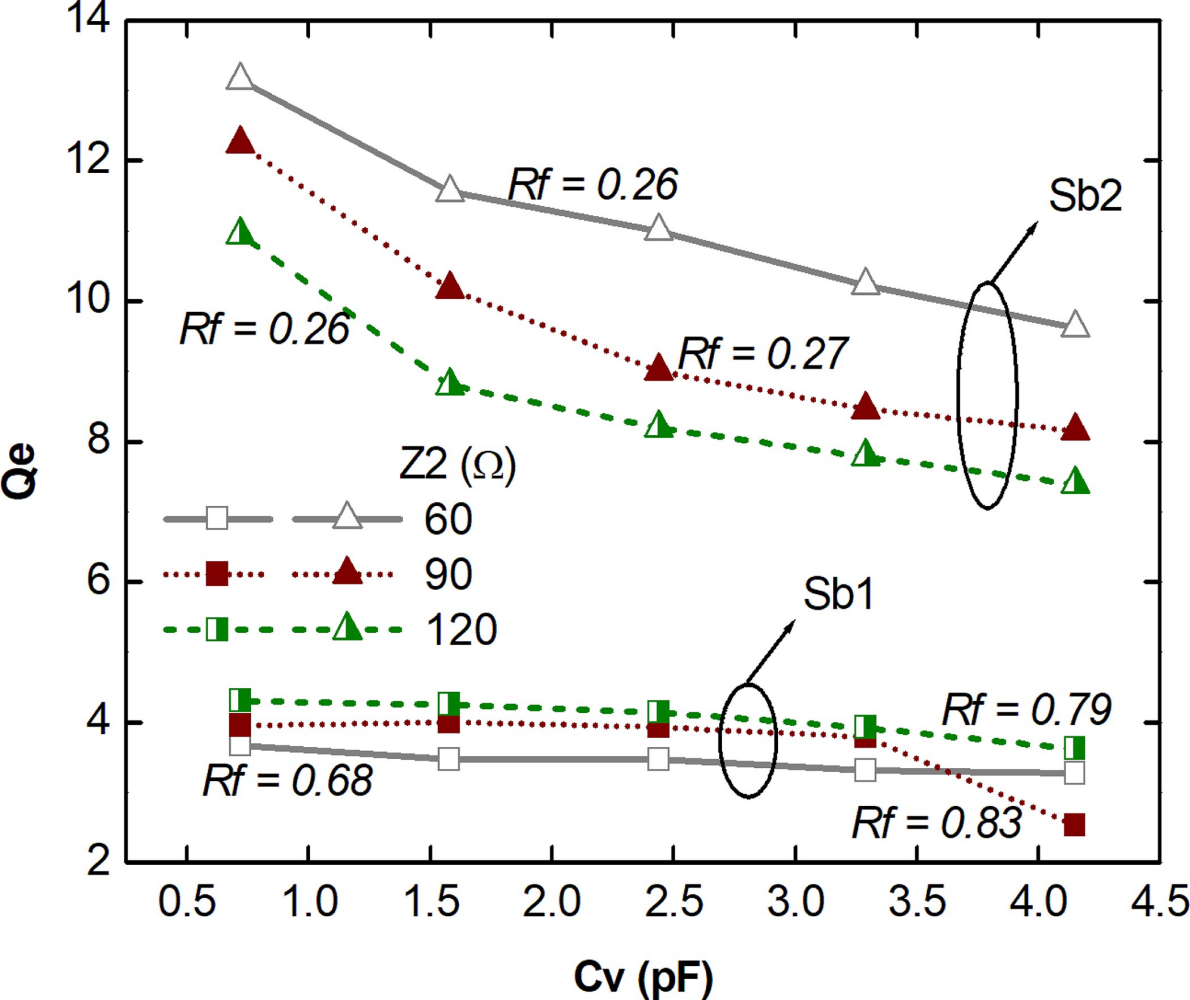
*Q*_*e*_ as function of Cv for Rb1 and Rb2, with different values for Z2.

Finally, a study was carried out on the external quality factor *Q*_*e*_ of the filter for both rejection ranges considering the variable capacitance *C*_*v*_ with values between 0.72 and 4.15 pF. Specific impedance levels were adopted for *Z*_2_, keeping the other impedances and physical parameters of the layout constant. Fig 6 presents the response of *Q*_*e*_ as a function of the variable capacitance *C*_*v*_ for the two rejection ranges Rb1 and Rb2. As analyzed in previous studies, the shift in frequency from the variable capacitance *C*_*v*_ results in a decrease in *Q*_*e*_ for both rejection ranges, being more pronounced for Rb2. However, the *Rf* levels for Rb1 (0.68, 0.83 and 0.79) remain much higher compared to Rb2 (0.26, 0.27 and 0.26). In general, higher levels of *Z*_2_ impedance result in lower *Q*_*e*_ values for both rejection ranges.

## 3. TBSF implementation

According to the objective of the work, which is to obtain a tunable band reject filter with operation in bands below 3.0 GHz, the impedances and consequently the dimensions of the layout presented in Fig 1 were optimized. According to the studies and analyzes carried out in the previous section, the following parameters were adopted for the TBSF: *Z*_1_ = 40.0 Ω, *Z*_2_ = 75.0 Ω, *Z*_3_ = 62.0 Ω. Regarding the length of the lines, the format described in Section 2 was preserved, with l1 = *λ*_0_/4, l2 = l3 = *λ*_0_/8, with *λ*_0_ being the wavelength at the resonance frequency *f*_0_, here adopted as being 0.60 GHz.

For the implementation of the layout in microstrip line technology, a modeling was initially adopted in Ansoft HFSS software in order to establish a previous response of the model and make adjustments to the geometry of the resonator through full-wave simulations. Considering a RO3006 dielectric substrate (*ε*_*r*_ = 6.15, tanδ = 0.0024, h = 1.52 mm), the physical dimensions chosen for the TBSF are presented in Table 1. The planar area of the TBSF layout is 0.17*λ*_*g*_ x 0.16*λ*_*g*_ mm^2^, where *λ*_*g*_ represents the guided wavelength at the frequency 0.60 GHz.

**Table 1:** Physical parameters of the proposed TBSF layout.

Parameter	Value (mm)	Parameter	Value (mm)	Parameter	Value (mm)
*L*_0_	5.58	*W*_0_	2.23	*W*_3_	1.20
*L*_1_	19.35	*W*_1_	3.25	*g*	3.0
*L*_3_	26.32	*W*_2_	0.70		

**Fig 7:**
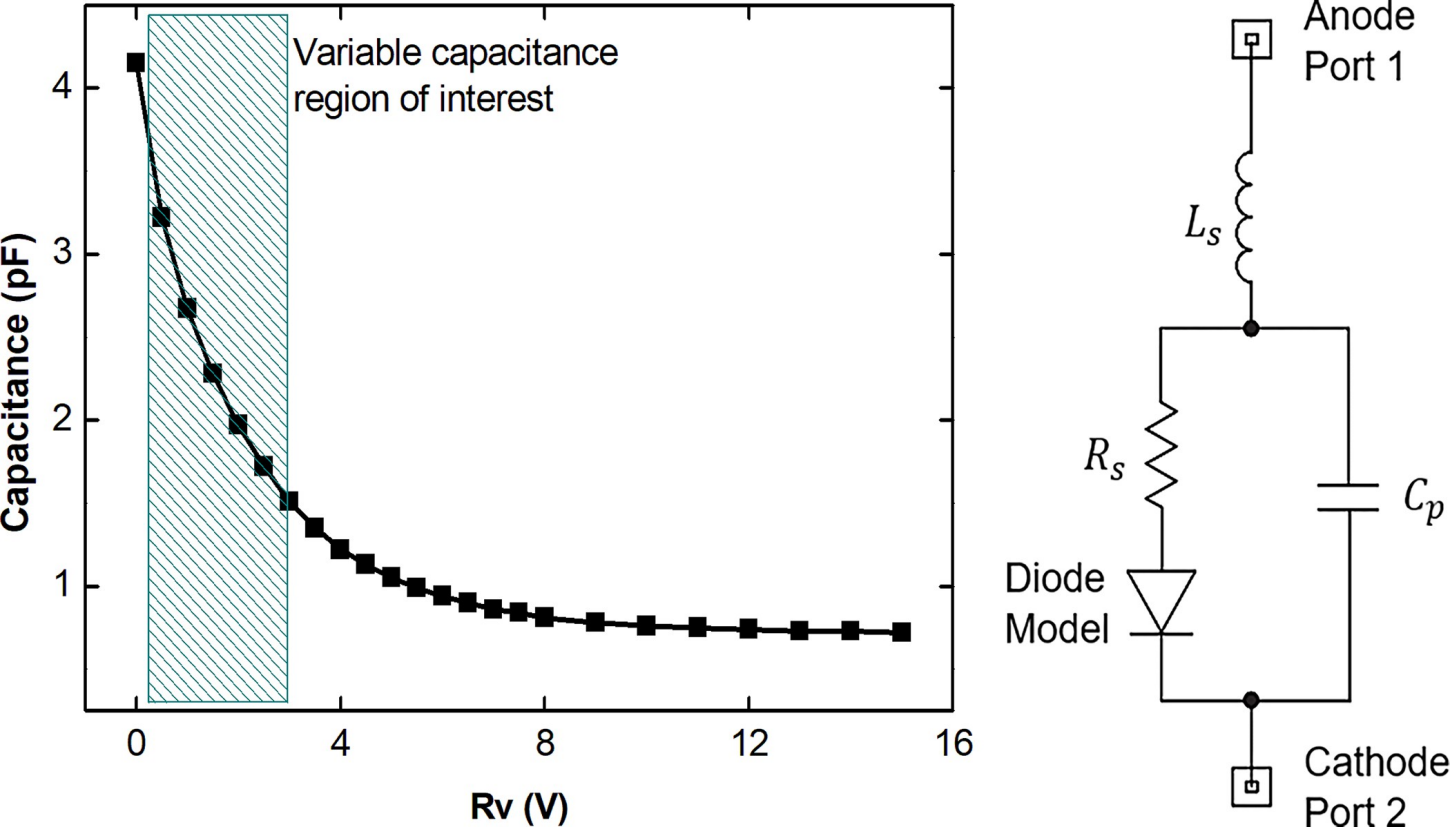
Parameters of Skyworks model SMV1232 varactor diode [33]. (a) capacitive response as a function of reverse bias. (In blue hatch, reverse polarization region and capacitance adopted in this work). (b) Simplified Equivalent Circuit of the SMV1232.

For the fabrication of the TBSF based on varactor diodes to implement the frequency tuning principle, the SMV1232 model from Skyworks [33] was chosen as the variable capacitance, whose capacitive response as a function of the reverse bias voltage Rv is shown in Fig 7(A). According to the manufacturer’s reference data, it is possible to apply Rv levels between 0 and 15.0 V, corresponding to a variable capacitive response between 0.72 and 4.15 pF. Due to constructive issues, a polarization level between 0.2 and 3.0 V is used, presenting a capacitive response between 1.58 and 4.05 pF. The simplified equivalent circuit of the varactor diode is shown in Fig 7(B), where Ls equals 0.7 nH and Rs equals 1.5 Ω the inductance and series resistance, respectively, of the device. Cp represents the capacitance whose value changes as a result of the reverse bias between the anode and cathode of the varactor diode. The ideal Diode Model demonstrates the common behavior of a diode depending on the level of polarization between the terminals.

**Fig 8:**
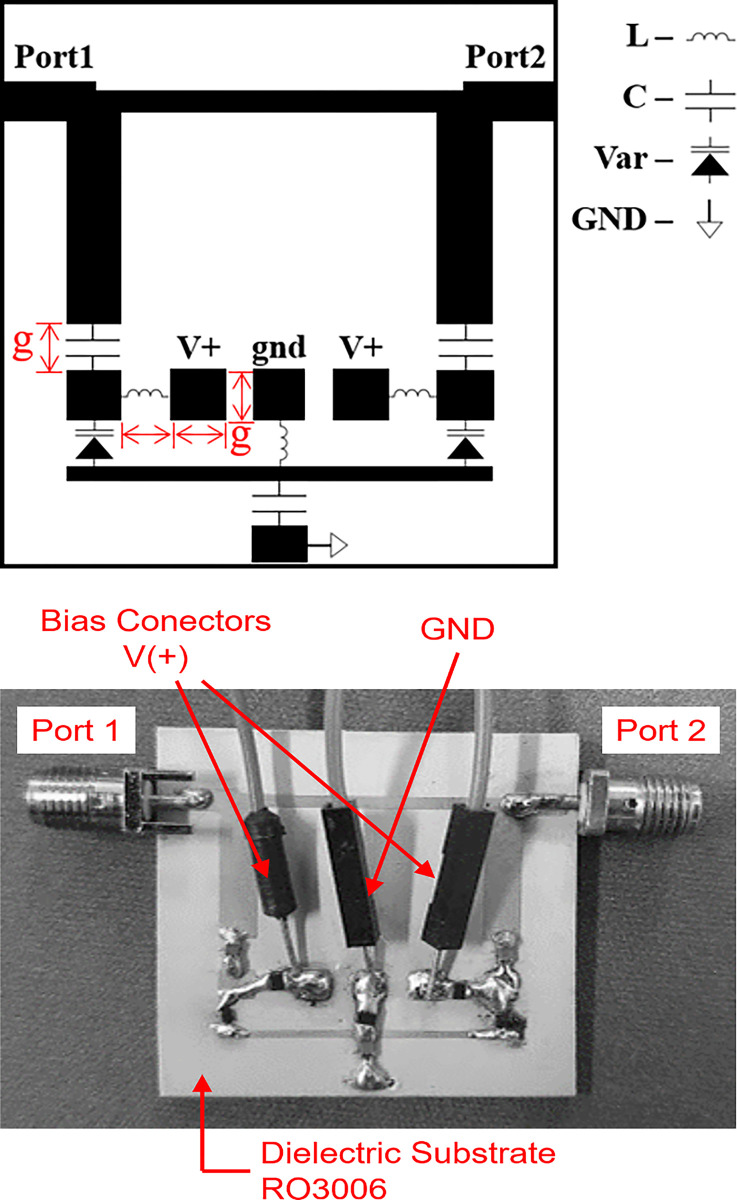
TBSF (a) layout with bias circuits and (b) fabricated prototype.

To establish a correct and safe form of polarization of the varactor diodes used in the TBSF, polarization circuits based on 0.1 μH bias inductors and 10.0 nF DC blocking capacitors were used, according to the layout shown in Fig 8(A), with components manufactured using SMD (surface-mounted device) technology. The voltage V(+) applied to the pads establishes the level of reverse voltage across the varactor diode, causing it to present the variable capacitance value *C*_*v*_ compatible with the frequency response required for the TBSF. In Fig 8(B) it is possible to observe the TBSF prototype fabricated in microstrip technology, highlighting the connectors responsible for the power supply. Next, the results from the laboratory measurement of the manufactured prototype are presented.

## 4. Results and discussion

**Fig 9:**
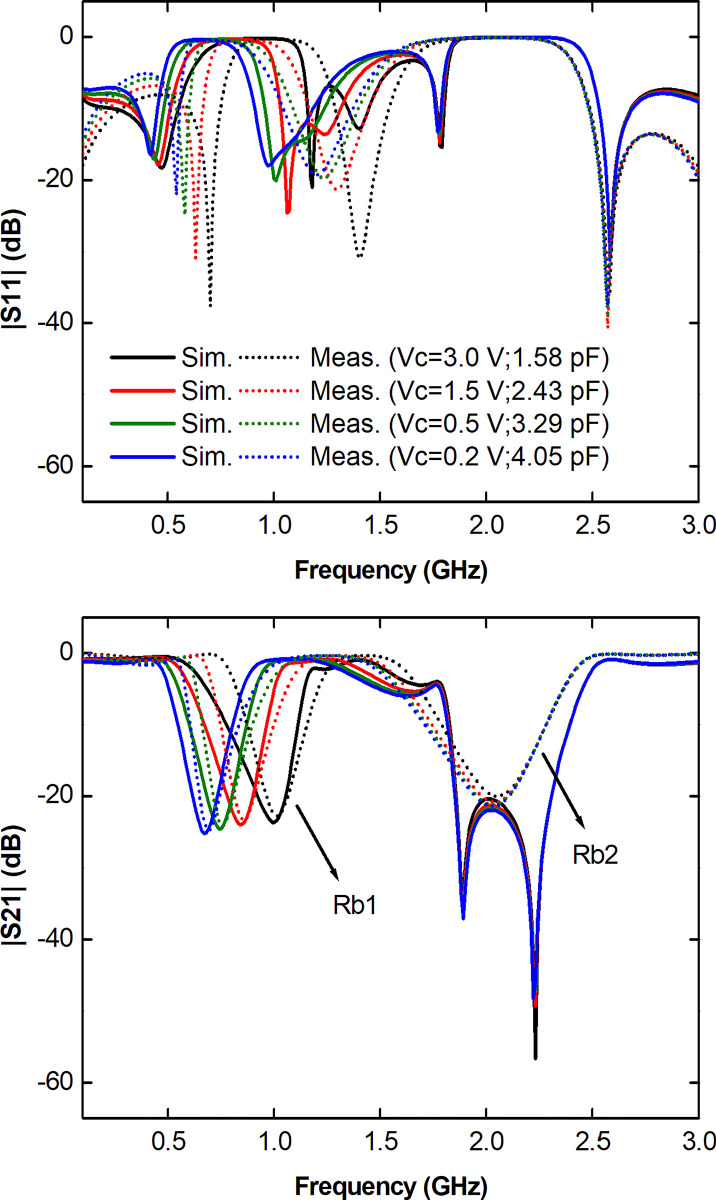
Comparison of simulated and measured results. (a) |S_11_| (dB). (b) |S_21_| (dB).

To validate the design procedure developed in the previous sections, a prototype was fabricated in microstrip technology (Fig 8(B)) and subjected to a routine of measurements in its ports 1 and 2 through the use of a vector network analyzer (VNA) miniVNA Tiny model from mRS Radio Solutions properly calibrated to an input impedance of 50.0 Ω on its two ports. Fig 9 presents the comparison of the curves derived from the frequency response obtained via numerical simulation and via measurement in the prototype with respect to the S-parameters |S_11_| and |S_21_| in the TBSF ports. To promote the change in the capacitive response of the set of varactor diodes used in the TBSF, specific Vc polarization voltage levels of 3.0, 1.5, 0.5 and 0.2 V were adopted, this margin corresponding to a capacitive amplitude between 1.58 and 4.05 pF. The variable capacitance resulting from the change in the reverse bias voltage across the pair of varactor diodes thus results in the change in the frequency response of the proposed TBSF.

As can be seen in Fig 9, the measured frequency response for the TBSF features two adjustable rejection bands (Rb1 and Rb2), with Rb1 between 0.6 and 1.15 GHz with a percent bandwidth at -3.0 dB variable from 27.0 to 36.0%, corresponding to a Tunning Range (*Rf*) of 0.63. As for Rb2, a tunable operation between 1.71 and 2.28 GHz is observed, with a BW of 24.0 to 28.0% and an *Rf* of 0.29. Both the first and second rejection bands have a rejection level above 20.0 dB across the band. Rb1 shows a larger change as a function of *C*_*v*_, as observed in the studies developed in the previous sections, with an operation below -3.0 dB being measured at 0.6 GHz with a reverse bias voltage of 0.2 V, while with a reverse voltage of 3.0 V, operation at 1.15 GHz is verified. With regard to Rb2, the data demonstrate greater stability in frequency in the face of a change in *C*_*v*_, as a consequence of the change in the reverse bias voltage Vc. These behaviors can be explained due to the process of manufacturing and building the prototype together with the discrete components. However, from the initial stages of analysis, it was possible to conclude that Rb1 was more influenced by the variable capacitance, while Rb2 was more stable.

**Fig 10:**
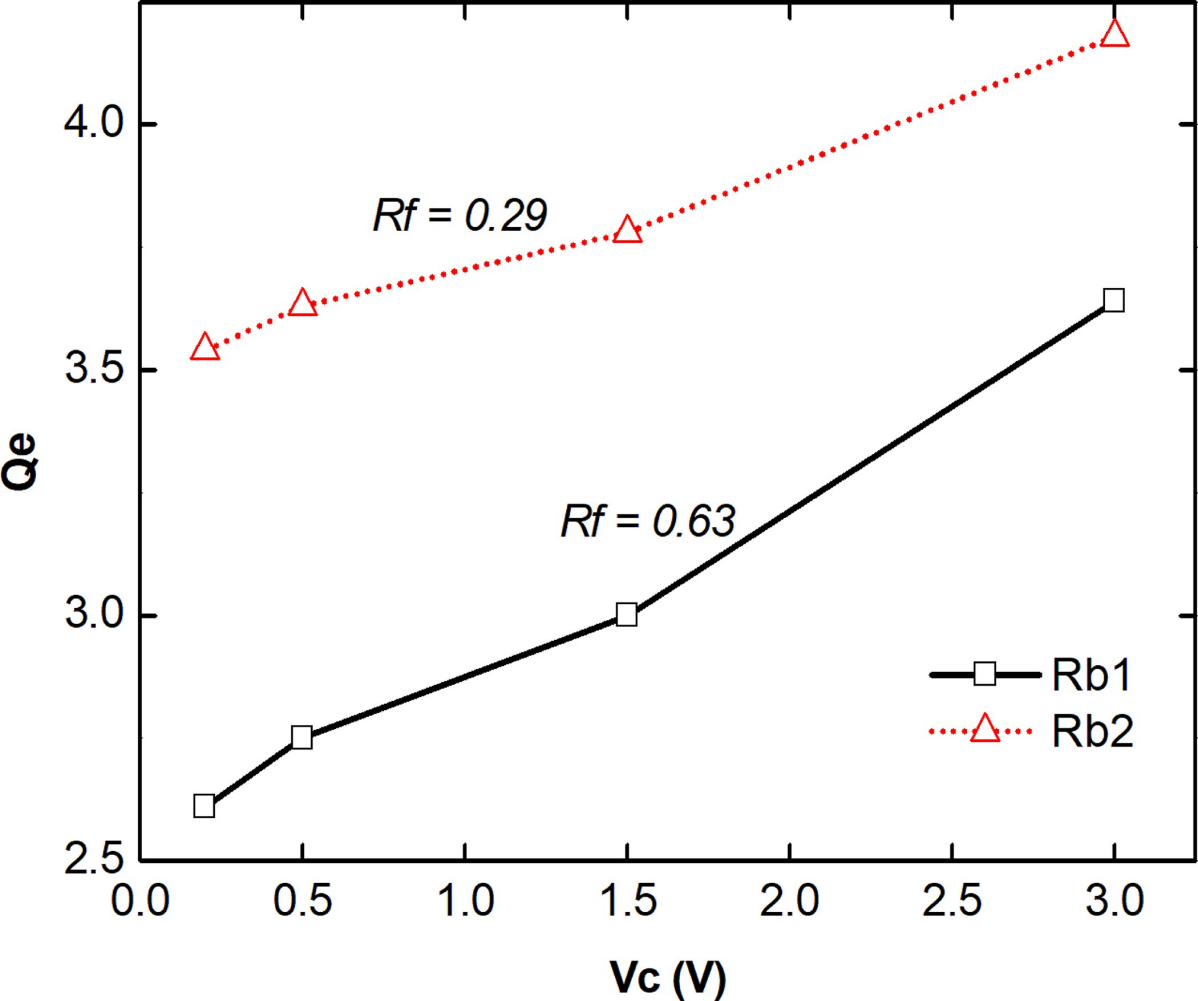
*Q*_*e*_ measured as function of Cv for Rb1 and Rb2.

Fig 10 presents experimental external quality factor *Q*_*e*_ results. The *Q*_*e*_ of the filter was analyzed as a function of the adjustment of the polarization voltage *C*_*v*_ for both rejection ranges. It is observed that for both bands, *Q*_*e*_ increased in face of the shift in frequency as a result of the Vc adjustment. Also, the *Rf* values were obtained for each range, with Rf1 equal to 0.63 much more adjustable compared to *Rf*2 equal to 0.29, according to the research presented above.

Table 2 presents a summary of the proposed TBSF results and a comparison with other tunable bandstop filters proposed in the literature, that operate in similar frequency bands. As it can be concluded, the proposed configuration has a performance comparable with the other configurations in terms of rejection level and size, but outperforms them in tunning range (Rf). Moreover, it requires a less complex design procedure.

**Table 2:** Comparison with previous tunable bandstop filters.

Reference	Resonator Topology	Frequency Operation (GHz)	Tunning Range-Rf (%)	Rejection Level (dB) in band	Size (λg x λg)	Design Complexity
[20]	Coupled lines	1.70–2.30	30.0	> 16.4	0.1152	High
[21]	Coupled lines	0.58–1.07	59.4	> 10.5	0.0900	Median
[22]	Stub-loaded	0.66–0.99	40.0	> 27.0	0.0190	High
[23]	Coupled lines/SIR	0.6–1.015	51.4	> 23.0	0.0361	Median
*This Work*	Square-ring	0.6–1.15	63.0	> 20.0	0.0272	Median
		1.71–2.28	29.0	> 20.0		

## 5. Conclusions

A tunable dual bandstop filter based on the use of varactor diodes is presented. The relationship between geometry input admittance and frequency response as a function of variable capacitance is investigated and discussed through the proposed analytical layout model and numerical electromagnetic simulations. Two key parameters are considered as a reference in the development and optimization, that is, the tuning range (*Rf*) and the external quality factor (*Q*_*e*_). A prototype was designed and fabricated using microstrip printed circuit board technology. The good agreement obtained between analytical and numerical simulations, and experimental results, validated the proposed design procedure and allowed the envisaged proof of concept. As a result, it was possible to obtain a bandstop filter model with two frequency tunable rejection bands through a polarization signal, the first in the range between 0.6 and 1.15 GHz with a tuning range of 63.0%, while the second rejection band demonstrated operation in the range 1.71–2.28 GHz with an *Rf* of 29.0%, making the reconfigurable model attractive for use in modern communication systems operating below 3.0 GHz.

## References

[1] Basit A, Khattak MI, Zubir F, Shah SW. Miniaturized ultra-wideband filter with independently controlled notch bands for 5.1/6/8 GHz wireless applications. PloS one. 2022;17: e0268886. doi: 10.1371/journal.pone.0268886 35679270 PMC9182340

[2] Al-Yasir YI, Ojaroudi Parchin N, Abd-Alhameed RA, Abdulkhaleq AM, Noras JM. Recent progress in the design of 4G/5G reconfigurable filters. Electronics. 2019;8: 114. doi: 10.3390/electronics8010114

[3] Zhang W, Feng F, Liu W, Yan S, Zhang J, Jin J, Zhang QJ. Advanced parallel space-mapping-based multiphysics optimization for high-power microwave filters. IEEE Trans. Microw. Theory Tech. 2021; 69: 2470–2484. doi: 10.1109/TMTT.2021.3065972

[4] Wang CH, Shi XM. Miniaturized tri-notched wideband bandpass filter with ultrawide upper stopband suppression. Sci. Rep. 202111: 1–10. doi: 10.1038/s41598-021-92394-7 34155281 PMC8217267

[5] Zhang G, Basit A, Khan MI, Daraz A, Saqib N, Zubir F. Multi Frequency Controllable In-Band Suppressions in a Broad Bandwidth Microstrip Filter Design for 5G Wi-Fi and Satellite Communication Systems Utilizing a Quad-Mode Stub-Loaded Resonator. Micromachines. 2023; 14: 866. doi: 10.3390/mi14040866 37421099 PMC10145329

[6] Fu W, Li Z, Liu P, Cheng J, Qiu X. Modeling and analysis of novel CSRRs-loaded dual-band bandpass SIW filters. IEEE Trans. Circuits Syst. II: Express Br. 2021;68: 2352–2356. doi: 10.1109/TCSII.2021.3052574

[7] Zaidi AM, Kanaujia BK, Khan T, Beg MT, Rawat K, Rambabu K, et al. Multiband Design Techniques for Passive Planar Microwave Circuits: A Review. IEEE Microw. Mag. 2022;23: 57–69. doi: 10.1109/MMM.2022.3180496

[8] Barik RK, Koziel S, Szczepansk S. Wideband Highly-Selective Bandpass Filtering Branch-Line Coupler. IEEE Access. 2022;10: 20832–20838. doi: 10.1109/ACCESS.2022.3152802

[9] Kumar KVP, Velidi VK, Rajkumar R, Rao TR. A Compact Ultra-Wideband Multi-Mode Bandpass Filter With Sharp-Rejection Using Stepped Impedance Open Stub and Series Transformers. IEEE Trans. Circuits Syst. II: Express Br. 2022; doi: 10.1109/TCSII.2022.3192512

[10] Abdul Rehman M, Khalid S, Mushtaq B, Idrees M. Design of a novel compact highly selective wideband bandstop RF filter using dual path lossy resonator for next generation applications. Plos one. 2022;17: e0273514. doi: 10.1371/journal.pone.0273514 36315491 PMC9621440

[11] Farzami F, Khaledian S, Stutts AC, Smida B, Erricolo D. Embedded Split Ring Resonator Tunable Notch Band Filter in Mi-crostrip Transmission Lines. IEEE Access. 2022;10: 37294–37304. doi: 10.1109/ACCESS.2022.3164699

[12] Kong M, Wu Y, Zhuang Z, Liu Y. Wideband bandstop filter with extreme sharp skirt selectivity. IEEE Microw. Wirel. Compon. Lett. 2018;28: 1104–1106. doi: 10.1109/LMWC.2018.2874130

[13] Liu L, Liang X, Fan H, Jin R, Bai X, Geng J. Compact wideband bandstop filter with directly controlled rejection. IEEE Trans. Circuits Syst. II: Express Br. 2021;68: 2282–2286. doi: 10.1109/TCSII.2021.3049693

[14] Gupta A, Chauhan M, Rajput A, Mukherjee, B. Wideband bandstop filter using L-shaped and Quad mode resonator for C and X band application. Electromagnetics. 2020;40: 177–185. doi: 10.1080/02726343.2020.1726003

[15] Islam H, Das S, Ali T, Kumar P, Dhar S, Bose T. Split ring resonator-based bandstop filter for improving isolation in compact mimo antenna. Sensors. 2021;21: 2256. doi: 10.3390/s21072256 33804815 PMC8037282

[16] Hussain M, Ali EM, Awan WA, Hussain N, Alibakhshikenari M, Virdee BS, et al. Electronically reconfigurable and conformal triband antenna for wireless communications systems and portable devices. Plos one. 2022;17: e0276922. doi: 10.1371/journal.pone.0276922 36454808 PMC9714694

[17] Awan W, Naqvi S, Ali W, Hussain N, Iqbal A, Tran H, et al. Design and realization of a frequency reconfigurable antenna with wide, dual, and single-band operations for compact sized wireless applications. Electronics. 2021;10: 1321. doi: 10.3390/electronics10111321

[18] Borah D, Gupta A, Kalkur TS, Miller K. A 5-Gb/s Adaptive Continuous Time Linear Equalizer Using Ferroelectric Capacitor. IEEE Trans. Compon. Packaging. Manuf. Technol. 2022;12: 647–654. doi: 10.1109/TCPMT.2022.3158933

[19] Bandyopadhyay A, Sarkar P, Ghatak R. A Bandwidth Reconfigurable Bandpass Filter for Ultra-Wideband and Wideband Applications. IEEE Trans. Circuits Syst. II: Express Br. 2022. doi: 10.1109/TCSII.2022.3167028

[20] Chen FC, Li RS, Chen JP. A tunable dual-band bandpass-to-bandstop filter using pin diodes and varactors. IEEE Access. 2018;6: 46058–46065. doi: 10.1109/ACCESS.2018.2862887

[21] Cai J, Yang YJ, Qin W, Chen JX. Wideband tunable differential bandstop filter based on double-sided parallel-strip line. IEEE Trans. Compon. Packaging. Manuf. Technol. 2018;8: 1815–1822. doi: 10.1109/TCPMT.2018.2794993

[22] Ebrahimi A, Baum T, Scott J, Ghorbani K. Continuously tunable dual-mode bandstop filter. IEEE Microw. Wirel. Compon. Lett. 2018;28: 419–421. doi: 10.1109/LMWC.2018.2821841

[23] Qin W, Cai J, Li YL, Chen JX. Wideband tunable bandpass filter using optimized varactor-loaded SIRs. IEEE Microw. Wirel. Compon. Lett. 2017;27: 812–814. doi: 10.1109/LMWC.2017.2734848

[24] Fan M, Song K, Zhu Y, Fan Y. Compact bandpass-to-bandstop reconfigurable filter with wide tuning range. IEEE Microw. Wirel. Compon. Lett. 2019;29: 198–200. doi: 10.1109/LMWC.2019.2892846

[25] Wu G, Wu H, Qin W, Shi J, Zhang W, Lin L, Li Q. Design of a Switchable Filter for Reflectionless-Bandpass-to-Reflectionless-Bandstop Responses. Micromachines. 2023;14: 424. doi: 10.3390/mi14020424 36838124 PMC9961987

[26] Zhibin Z, Lei B. Frequency-reconfigurable wideband bandstop filter using varactor-based dual-slotted defected ground structure. IEICE Electron. Express. 2021;18: 20210154–20210154. doi: 10.1587/elex.18.20210154

[27] Chen R, Zhang Q, Zhou L, Chen C. Reconfigurable Dual-Band Bandpass-to-Bandstop Filter Using SAW Resonators and Lumped Elements. In 2021 IEEE MTT-S International Wireless Symposium (IWS). 2021:1–3. IEEE. doi: 10.1109/IWS52775.2021.9499428

[28] Lee T, Laurin J, Wu K. Reconfigurable filter for bandpass-to-absorptive bandstop responses. IEEE Access. 2020;8: 6484–6495. doi: 10.1109/ACCESS.2019.2963710

[29] Ren B, Ma Z, Liu H, Guan X, Wang X, Wen P, Ohira M. Differential dual-band superconducting bandpass filter using multimode square ring loaded resonators with controllable bandwidths. IEEE Trans. Microw. Theory Tech. 2018;67: 726–737. doi: 10.1109/TMTT.2018.2882487

[30] Pozar DM. Microwave engineering. John wiley & sons. 2011. 9781522575405.

[31] Ozaki H, Ishii J. Synthesis of a class of strip-line filters. IRE Transactions on Circuit Theory. 1958;5: 104–109. doi: 10.1109/TCT.1958.1086441

[32] Nemoto Y, Kobayashi K, Sato R. Graph transformations of nonuniform coupled transmission line networks and their application. Trans Microw Theory Tech. 1985;33: 1257–1263. doi: 10.1109/TMTT.1985.1133208

[33] Skyworksinc.com. Skyworks—Products Details. 2022. [online] Available at: https://www.skyworksinc.com/Products/Diodes/SMV1232-Series.

